# Artificial neural network approach for selection of susceptible single nucleotide polymorphisms and construction of prediction model on childhood allergic asthma

**DOI:** 10.1186/1471-2105-5-120

**Published:** 2004-09-01

**Authors:** Yasuyuki Tomita, Shuta Tomida, Yuko Hasegawa, Yoichi Suzuki, Taro Shirakawa, Takeshi Kobayashi, Hiroyuki Honda

**Affiliations:** 1 Department of Biotechnology, School of Engineering, Nagoya University, Furo-cho, Chikusa-ku, Nagoya 464-8603, Japan; 2 Department of Medical Genetics, Tohoku University School of Medicine, 1-1 Seiryou-machi, Aoba-ku, Sendai, 980-8575, Japan; 3 Department of Health Promotion and Human Behavior, Kyoto University School of Public Health, Yoshida-Konoe cho, Sakyo-ku, Kyoto, 606-8501, Japan

## Abstract

**Background:**

Screening of various gene markers such as single nucleotide polymorphism (SNP) and correlation between these markers and development of multifactorial disease have previously been studied. Here, we propose a susceptible marker-selectable artificial neural network (ANN) for predicting development of allergic disease.

**Results:**

To predict development of childhood allergic asthma (CAA) and select susceptible SNPs, we used an ANN with a parameter decreasing method (PDM) to analyze 25 SNPs of 17 genes in 344 Japanese people, and select 10 susceptible SNPs of CAA. The accuracy of the ANN model with 10 SNPs was 97.7% for learning data and 74.4% for evaluation data. Important combinations were determined by effective combination value (ECV) defined in the present paper. Effective 2-SNP or 3-SNP combinations were found to be concentrated among the 10 selected SNPs.

**Conclusion:**

ANN can reliably select SNP combinations that are associated with CAA. Thus, the ANN can be used to characterize development of complex diseases caused by multiple factors. This is the first report of automatic selection of SNPs related to development of multifactorial disease from SNP data of more than 300 patients.

## Background

In recent years, the number of patients suffering from allergic asthma has increased [1], and allergic diseases including asthma have become a social problem affecting medical costs and quality of life. Allergic asthma is a complex disorder characterized by airway inflammation, bronchial hyperresponsiveness and reversible airway obstruction. Elevated numbers of activated Th2 cells, mast cells and eosinophils in the bronchial mucosa cause certain features of asthma, including increased serum IgE levels in allergic asthma. The available data suggest that there are many potential susceptible genes for allergic asthma, including genes for cytokines, receptors, transcription factors, immune recognition and regulation of lipid mediator generation. A few susceptible genes for allergic asthma have been identified that may be associated with the asthmatic phenotype [2-4], but definite susceptible genes have not been identified yet. Thus, large-scale analysis of gene markers is needed, along with identification of association between these genetic polymorphisms and the asthmatic phenotype, and its development mechanism. It has been reported that the human genome has 3 to 10 million single nucleotide polymorphisms (SNPs). A SNP in a coding region can cause amino acid substitution, resulting in functional modification of the protein; a SNP in a promoter region can affect transcriptional regulation; and a SNP in an intron region can affect splicing and expression of the gene. Thus, SNPs can be highly informative for identifying genetic factors of multifactorial disease such as allergic asthma.

In the present study, we analyzed associations between SNPs and childhood allergic asthma (CAA), which is more strongly influenced by genetic factors than other types of allergic asthma. We performed this analysis using an artificial neural network (ANN), which is a computer-based algorithm that can be trained to recognize and categorize complex patterns [5-8]. ANNs have been used for discrimination between subtly different clinical disease lesions; e.g., premalignant lesion Barrett's versus esophageal cancer, based on microarray data [6]. In a previous study, we performed severity assessment of senile dementia of Alzheimer type using ANN modeling of electroencephalogram data. The average error of the ANN model for assessment scale (HDS-R) score was 2.64 points out of 30 [7]. We have also used an ANN for prediction of 4 allergic diseases using SNP data [8]; 82 subjects with data for 6 SNPs were analyzed, and the ANN model predicted diagnosis with accuracy of more than 78%. Thus, we have achieved sufficiently high accuracy with ANNs using relatively little SNP data.

**Figure 1:**
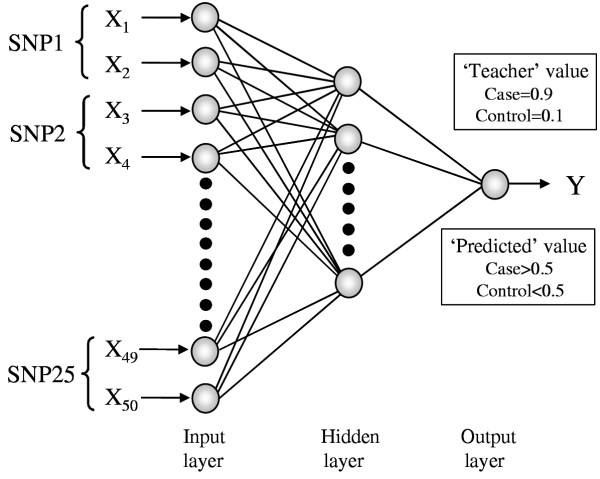
Structure of ANN. For the analysis of 25 SNPs, 50 input layer units were provided. The number of hidden layer units was changed from the usual 6 to 10, to optimize the ANN for the highest possible prediction accuracy. The output layer had only 1 unit. Because the ANN model has connection weight parameters, which depend on the number of connection units, analysis of 25 SNPs with 6 hidden layer units requires 306 connection weight parameters.

Here, we propose an ANN model (its structure is shown in Figure 1) suitable to diagnostic prediction of 172 subjects with CAA and 172 healthy subjects, using 25 SNPs in 17 genes shown in Table 1. For comparison with ANN, we also used logistic regression (LR) analysis, which is currently used to analyze medical statistics and equivalent to ANN with a single hidden node [9]. In order to selectively identify susceptible SNPs, a susceptible marker-selectable ANN is proposed, in which a parameter decreasing method (PDM) is incorporated. Information on obtaining the execute code, example data and documentation of this software is available at . Associations between combinations of important SNPs and CAA pathogenesis were investigated. A χ^2 ^test was performed for all 2-SNP and 3-SNP combinations.

## Results

### SNPs selected for diagnostic prediction with ANN

Several reports have suggested linkage between asthma and chromosomes. For example, genes in the 5q31-5q33 region code for Th2-type cytokines (IL-4, IL-13, which regulate B cell heavy-chain class-switching to IgE production) [10] and ADRB2 (which mediates airway smooth muscle relaxation and protects against bronchial hyperreactivity) [11,12]. IL-4 operates via the IL-4 receptor (IL-4R), which is encoded by a gene in chromosomal region 16q12. Mice deficient in the IL-4Rα chain lack IgE production and Th2 inflammatory reactions, and it has been shown that total IgE level is dependent on Ile50Val substitution [13]. In the present study, we analyzed 25 SNPs (Table 1) in 17 genes known to be associated with development of asthma. Association between these SNPs and CAA was assessed by *P*-value. As shown in Table 1, 21 of these 25 SNPs had a *P*-value greater than 0.1. The *P*-values of *CysLT2 *(108 C/A), *IL-4Rα *(148 G/A), *ADRB2 *(265 A/G) and *C5 *(4266 G/A) were 0.0036, 0.0155, 0.0541 and 0.0581, respectively. When *CysLT2 *(108 C/A), which had the lowest *P*-value of 25 SNPs, was used for discrimination between case and control as a sole factor, prediction accuracy was 54.4%, and the sensitivity and specificity was 12.8% and 95.9%, respectively, compared with the number of case and control subjects to assess discrimination performance (genotype CC; case 150, control 165, genotype CA or AA; case 22, control 7). Thus, we constructed a susceptible marker-selectable ANN model, which can discriminate between cases and controls using the selected susceptible SNPs, and which can include the association between combinations of SNPs and development of CAA.

### Diagnostic prediction using 25 SNPs

**Figure 2:**
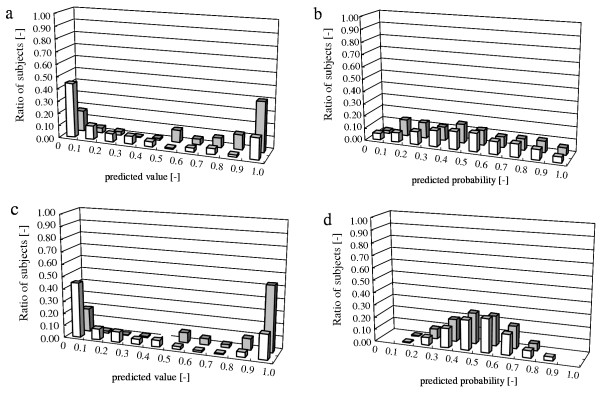
Diagnostic prediction of ANN (a) and LR (b) using 25 SNPs, and prediction of ANN (c) and LR (d) using the 10 selected SNPs. Prediction results of evaluation data are presented. Gray and white bars represent frequency of case subjects and control subjects, respectively.

We used a three-layered ANN with input, hidden and output layers (Figure 1). An ANN model and LR model, with 25 SNPs as input variables, were constructed with learning data, and we performed diagnostic prediction with evaluation data. The results of diagnostic prediction are shown in Figure 2a,2b and Table 2a. The ANN had higher prediction accuracy than LR. Accordingly, sensitivity and specificity, with both evaluation data and learning data, were higher for the ANN than for LR (Table 2a). In LR analysis, Monte Carlo study was performed to evaluate the effect of number of events per variable (EPV) [14]. It suggested that at least 10 events per variable analyzed were desirable to maintain the validity of the model. In the present study, we used 172 events per group and 51 variables (25 SNPs and 1 teacher value). LR would not have an enough power for parameter selection, because 172 events per group is small compared with that of variable. The construction of optimized LR model should be furthermore investigated.

### Selection of susceptible SNPs for CAA

**Figure 3:**
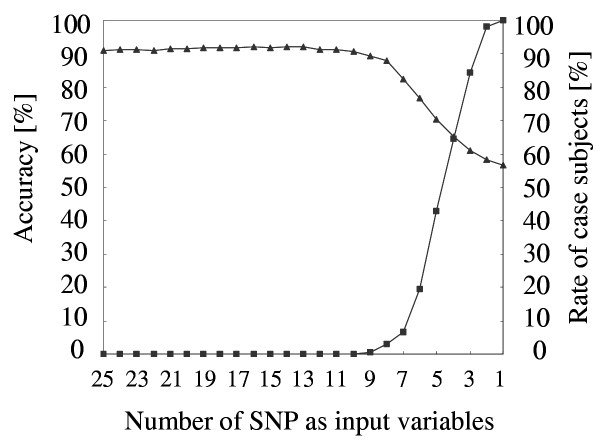
Effect of number of input variables on ANN model accuracy during PDM procedure. Closed triangles represent average accuracy for learning and evaluation data. Closed squares represent the rate of case subjects that means *N'*_*case*_/*N*_*case*_. In this case, *N'*_*case *_is the number of cases whose genotype pattern is match to control's genotype pattern at least one control (*N*_*case *_= 172 subjects).

Ritchie *et al*. [5] reported the optimization of the architecture using genetic programming neural networks (GPNN) [5]. If important SNPs were previously determined, optimization of network architecture should be carried out. Genetic programming neural networks have contributed the construction of ANN model with high performance. In the present study, however, many candidate SNPs were used and the selection of SNPs is firstly desired. Therefore, in order to extract SNPs closely associated with CAA, we tried optimization of input variables by PDM in the ANN model, while the architecture of a neural network was not modified. Five PDM trials were performed. Figure 3 shows typical results for change of accuracy during PDM procedure. When input variables were excluded one by one to preserve prediction accuracy (as described in Methods), the accuracy began to decrease after the number of SNPs used for modeling reached 10. When the number of SNPs used for modeling decreased, coincidence of genotyping pattern between cases and controls inevitably occurred. When genotyping pattern of a case was coincident with that of controls, the learning for model construction did not progress well. We investigated the rate of case subjects whose genotype patterns were coincident with that of control subjects at each step of PDM (Figure 3). Rate of case subjects [%] in Figure 3 means *N'*_*case*_/*N*_*case*_. In this case, *N'*_*case *_is the number of cases whose genotype pattern is match to control's genotype pattern at least one control (*N*_*case *_= 172 subjects). As shown in Figure 3, there was little coincidence of genotype patterns when more than approximately 7 SNPs were used in ANN modeling. Therefore, the decrease in accuracy was considered to be due to omission of a highly important SNP. The remaining 10 SNPs were worth investigating as important factors.

**Figure 4:**
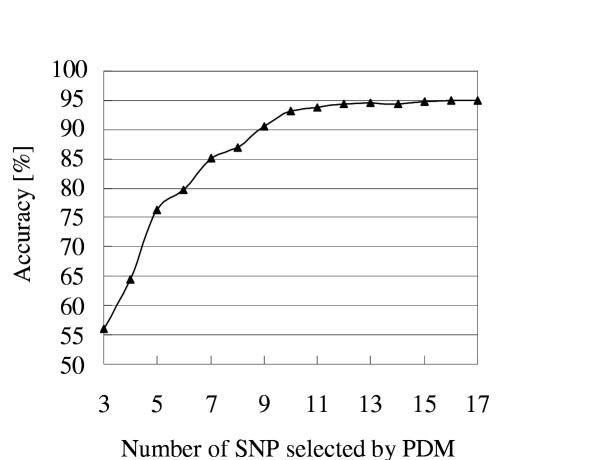
Reconstruction of ANN model using SNPs listed in Table 3. Closed triangles represent average accuracy for all data of learning and evaluation.

To investigate the important SNPs, we counted the number of SNPs that remained within the last 10 input variables in 5 trials. The significance order of remaining SNPs was listed, and a score of order ranging from 1 to 10 points was determined, based on the significance order. The remaining SNPs were reordered according to sums of scores, as shown in Table 3. We believe that SNPs with higher scores are more important for development of CAA, because significance of SNPs correlated with the order of elimination via the PDM procedure described in methods section. ANN models were reconstructed using SNPs listed in Table 3. The number of input SNPs varied from three (*IL-4Rα *(148 G/A), *CysLT2 *(2534 A/G) and *IL-10 *(-571 C/A)) to 17 SNPs (all listed SNPs) according to the order of Table 3. When more than 10 SNPs were used as input variables, average accuracy for learning and evaluation data was high (Figure 4), and was almost equal to that of the model using 25 SNPs. These results suggest that the 10 SNPs selected in Table 3 are very important for prediction of development of CAA.

The results of diagnostic prediction using the 10 SNPs selected by PDM are shown in Figure 2c, and the accuracy, sensitivity and specificity are shown in Table 2b. In the ANN model, the accuracy, sensitivity and specificity with evaluation data were again sufficiently high, and were somewhat similar to the results from the analysis using 25 SNPs, although the number of input variables was markedly smaller than in the analysis using 25 SNPs. In particular, sensitivity was significantly high (77.9%), indicating that case subjects were more correctly diagnosed by this model. To compare with the LR model, LR model consisting of 10 SNPs selected by ANN was constructed (Figure 2d). As shown in Table 2b, the LR model constructed showed low accuracy. This result indicates high performance of ANN modeling for CAA prediction although selected SNPs would not be suitable for LR analysis. We concluded that the ANN model constructed with 10 SNPs could discriminate between cases and controls as precisely as the model constructed with 25 SNPs.

### Interaction between SNP and another SNP for CAA

To understand the importance of the 10 SNPs selected, we analyzed combinations of these 10 SNPs. We paid particular attention to SNP combinations associated with CAA, and assessed whether any combinations consisting of SNPs selected by ANN were associated with CAA. The relationships between 2-SNP or 3-SNP combinations and CAA development were examined by calculating *P*-value using the χ^2 ^test. In models using 10 SNPs selected by PDM or the other 15 SNPs, the total number of 2-SNP combinations and 3-SNP combinations (*N*_*comb*_) is 90 (_10_P_2_) or 210 (_15_P_2_), and 360 (_10_P_3_/2) or 1365 (_15_P_3_/2), respectively. With respect to 2-SNP combination between the SNP of interest and SNP A, *P*-value was calculated as follows. When patients were limited with certain pattern of another SNP, such as AA major homozygote of SNP A, patient distribution of the SNP of interest was investigated. With respect to 3-SNP combination, between the SNP of interest, and SNP A and B, *P*-value was calculated as follows. When patients were limited with certain pattern of two other SNPs, such as AA major homozygote of SNP A and BB major homozygote of SNP B, patient distribution of the SNP of interest was investigated.

To evaluate *P*-value of the combination, the usual Bonferroni correction of *P*-values was first investigated. To select the 2-SNP combination accompanied with minimum false positive, the criterion was *P *< 0.05/300. Here 300 cases correspond to _25_C_2_. Under this severe condition, there were no significant SNPs. As the same as 2-SNP combination, any significant combination was not obtained on 3-SNP combination under the threshold of *P *< 0.05/2300. Next, to determine important combination, *P*-value without Bonferroni correction was used, that is *P *< 0.05. Results are shown in Table 4. In 2-SNP combination, there were 13 combinations with *P *< 0.05 among total 90 combinations. In the case of 3-SNP, 72 combinations with *P *< 0.05 were existed in 360 exhaustive combinations. However, combinations possibly include several false positive significant combinations. Therefore, we paid attention to the SNP, of which *P*-value effectively decreases by combining with genotype or allele of other SNPs. We defined effective combination value (ECV). ECV2 or ECV3 is the ratio of 2 or 3-SNPs *P*-value to the product of each *P*-value. ECV is not indicator for avoiding false positives but for evaluation of interaction. For example, in 2 SNP combinations, when patients were limited with certain pattern of another SNP, such as AA major homozygote of SNP A, patient distribution of the SNP X of interest is investigated (*P *= *P*_*ax*_). If the 2-SNP combination is independent (no interaction) each other, *P*_*ax *_equals multiplication of *P*_*a *_and *P*_*x*_. ECV<1 means that the 2-SNP combination is not independent and two SNPs have any interaction each other.

The effect of ECV on number of effective combinations is shown in Table 4. About half number of 2-SNP combination satisfied the condition ECV2<1 (*N*_*ECV*2<1 _= 47). Among 13 combinations with *P *< 0.05 mentioned above, 11 combination also satisfied the same condition (*N*_*ECV*2<1,*P *_= 11). When ECV2<0.5, *N*_*ECV*2<0.5 _decreased the number to 27 and 10 of *N*_*ECV*2<1,*P *_= 11 still remained. When ECV2<0.1, *N*_*ECV*2<0.1 _became small and it was thought that positive combination may be lost. In the case of 3-SNP combination, only 20% of the total combination satisfied the condition *P *< 0.05. The combinations of 12% among the total combinations (43 combinations) satisfied ECV3<1. All of these 43 combinations also satisfied the condition *P *< 0.05. From these results, it was concluded that *P *< 0.05 is not strict criterion for 3-SNP combination analysis. In the case of 2-SNP combination, ECV2<0.5 was adequate as a selection of effective combination, because 77% of the combination with *P *< 0.05 still remained. From these consideration, we selected these two evaluation bases (*P*-value and ECV) in order to determine effective combinations and the combination with *N*_*ECV*<0.5,*P *_(*P *< 0.05 and ECV<0.5) was picked up. The combinations were used for the following investigation. The number of combination which satisfies the condition, *P *< 0.05 and ECV2<0.5 in 2-SNP combination and *P *< 0.05 and ECV3<0.5 in 3-SNP combination was designated as *N*_*ef*_, the number of effective combination, respectively (Table 5).

It is very important to clearly determine whether effective combinations frequently occur among groups of 10 SNPs selected by ANN modeling with PDM. It would be difficult to investigate the phenotypes associated with each of such a large number of combinations. Identifying effective 2-SNP combinations using the conditions described above is a useful method of identifying 2-SNP combinations that merit further investigation. Ten effective combinations were found among the 10 SNPs selected by ANN; 23 effective combinations were found between those 10 SNPs and the remaining 15 SNPs; and 3 effective combinations were found among the remaining 15 SNPs. It is likely that the former 10 combinations are more important than the latter 26 combinations, because the ANN model constructed using only the selected 10 SNPs exhibited sufficiently high accuracy to predict development of CAA. Susceptible genes for development of a multifactorial disease like CAA can correctly classify many subjects as cases or controls, and it is very important that those genes involve SNP combinations that have important interaction with high concentration ratio. We defined the concentration ratio as the ratio of *effective rate *to *random selection rate*. When the effective rate, *N*_*ef*_/Σ*N*_*ef*_, was calculated, it was found to be 0.28 (10/36) for the 10 SNPs selected by PDM, 0.64 (23/36) for combinations between the 10 selected SNPs and the remaining 15 SNPs, and 0.08 (3/36) for combinations of the remaining 15 SNPs (Table 5). The random selection rate, *N*_*com*_/_15_P_2 _shown in Table 5, represents the rate which the combination is selected from all 2-SNP combinations independently, 0.15 (90/_15_P_2_) for the 10 SNPs selected by PDM, 0.5 (300/_15_P_2_) for combinations between the 10 selected SNPs and the remaining 15 SNPs, and 0.35 (210/_15_P_2_) for combinations of the remaining 15 SNPs (Table 5). The concentration ratio was found to be 1.85 for the 10 SNPs selected by PDM, 1.28 for combinations between the 10 selected SNPs and the remaining 15 SNPs, and 0.24 for combinations of the remaining 15 SNPs (Table 5). The concentration ratio was higher for combinations among the 10 selected SNPs than for other combinations, so we can select 2-SNP combinations associated with CAA with high rate. The results are shown in Table 6.

In the next step, 3-SNP combinations were analyzed. The effective rate, the random selection rate, and the concentration ratio were calculated as well as the case of 2-SNP combination (Table 5). It was found to be 2.28 for each of the 10 selected SNPs alone (3:0 in Table 5), 1.33 for 2 of the 10 selected SNPs and 1 of the remaining 15 SNPs (2:1 in Table 5), 0.67 for 1 of the 10 selected SNPs and 2 of the remaining 15 SNPs (1:2 in Table 5), and 0.94 for the remaining 15 SNPs alone (0:3 in Table 5). The concentration ratio was higher for combinations among the 10 selected SNPs than for other combinations, so we can select 3-SNP combinations associated with CAA with high rate. The combination with the lowest ECV3 consisted of the genes *IL-4Rα*, and *C3 *(0.03526). This is about 3% of the value multiplied each *P*-value of 2-SNP combination (0.5060). For patients with genotype GA of *IL-4Rα *(148 G/A: Val50Ile) and genotype CT of *C3 *(4896 C/T), patient frequency against genotype of *C3 *(1692 G/A) had a *P*-value of 0.01784. For *C3 *(1692 G/A) alone, a *P*-value of 0.6993 was obtained, which was 40 times greater than the *P*-value of the 3-SNP combination. Thus the rate of correct identification of effective combinations evaluated by adjusted *P*-value and ECV selected based on PDM trials was higher than the corresponding randomized rate, implying that the ANN can reliably select SNP combinations that are associated with CAA.

**Figure 5:**
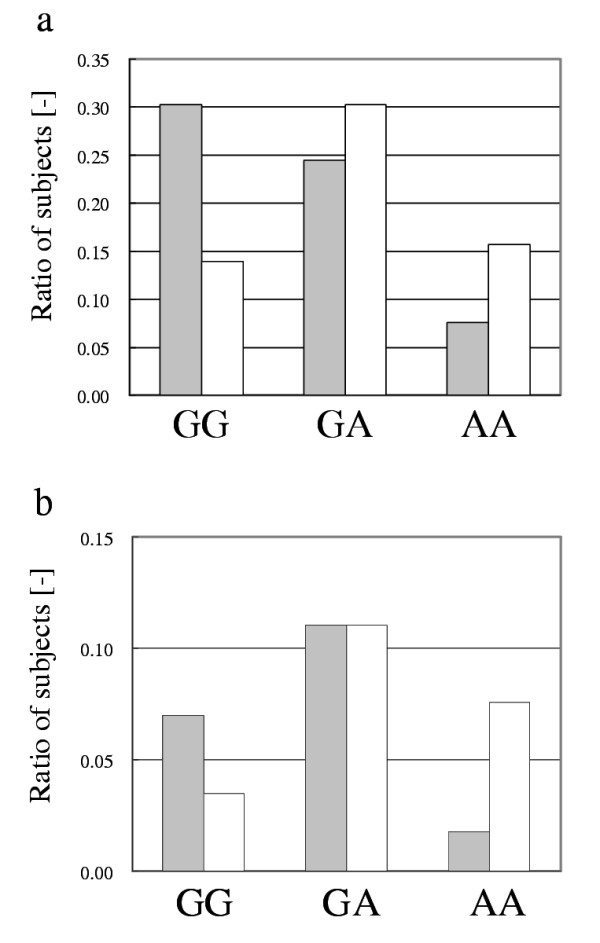
Distribution of *IL-4Rα *(148 G/A) genotype with *CysLT2 *(2534 A/G) genotype AG or GG (*P *= 0.00030) (a), and distribution of *C3 *(1692 G/A) genotype with *IL-10 *(-571 C/A) genotype CA and *IL-4 *(-590 C/T) genotype CT (*P *= 0.00426) (b). Gray and white bars represent frequency of case subjects and control subjects, respectively.

The 2-SNP combinations with the conditions described above among selected 10 SNPs are shown in Table 6. For example, in Table 6, for combinations between *CysLT2 *(2534 A/G) and *IL-4Rα *(148 G/A: Val50Ile), among subjects with a *CysLT2 *(2534 A/G) genotype of AG or GG (CAA, 107 subjects; healthy controls, 103 subjects), there was an important correlation with *IL-4Rα *(148 G/A: Val50Ile) genotype of GG, GA, AA (*P *= 0.00030). We examined the distributions of important combinations among subjects. A total of 52 CAA subjects and 24 healthy controls had genotype AG or GG at *CysLT2 *and genotype GG at IL-4R*α *(148 G/A) (Figure 5a).

The present findings also indicate that the 3-SNP combination consisting of *IL-10 *(-571 C/A), *IL-4 *(-590 C/T) and *C3 *(1692 G/A) is a susceptible factor of CAA (*P *= 0.00426). No association with CAA was found for any of these 3 SNPs alone (*P *= 0.1074, 0.9085, 0.6993, respectively; Table 1) or for any 2-SNP combinations of them (*P *= 0.1851, and 0.3002, respectively). Subjects with genotype CA at *IL-10 *(-571 C/A), genotype CT at *IL-4 *(-590 C/T) (CAA, 34 subjects; healthy controls, 38 subjects) and genotype GG at *C3 *(1692 G/A) (CAA, 12 subjects; healthy controls, 6 subjects) were estimated to be at high risk for pathogenesis of CAA. Furthermore, among the subjects with the same genotype pattern, the number of subjects with genotype AA at *C3 *(1692 G/A) were CAA, 3 and healthy controls, 13, respectively (Figure 5b).

Other remarkable combinations shown in Table 6 were also found among the 10 selected SNPs. For example, the number of cases with GG genotype at *IL-4Rα (*148G/A) and TT genotype at *C3 *(4896C/T) was 4 times the number of controls with that genotype combination (CAA, 20 subjects; healthy controls, 5 subjects) (*P *= 0.00271). There are no previous reports of association between these genotype combinations and CAA. The combination of *IL-4Rα *(148 G/A: Val50Ile) and *IL-4 *(-590 C/T) was also associated with CAA (*P *= 0.00689); association between allergic asthma and this combination has previously been reported [15,16].

## Discussion

To characterize the development mechanism, we investigated several relationships between SNPs and development of CAA, referring to previous papers, as described below. IL-4 is produced by Th2 cells, and exerts its activity by interacting with the receptor IL-4Rα, located on the surface of B cells. It has been reported that the V50 (148G)/R551(1827G) combination of *IL-4Rα *polymorphisms may be associated with enhancement of IL-4Rα function [16]. As concerns the polymorphisms on *IL-4*, it was reported that the -590T allele increases the strength of the *IL-4 *promoter compared with the -590C allele [15]. C3 is a proinflammatory mediator that binds to specific cell surface receptors and causes leukocyte activation, smooth muscle contraction and vascular permeability [17]. *C3*-deficient mice challenged with allergen show diminished airway hyperresponsiveness and lung eosinophilia, with dramatic reduction of the number of IL-4-producing cells and attenuation of IgE responses [18]. In the present study, we found that interaction between genotype TT at *C3 *(4896 C/T) and genotype GG at *IL-4Rα *(148 G/A) may be associated with CAA, but details of interaction between these polymorphisms combinations and development mechanisms have not been clarified. The present findings indicate that, among subjects with an *IL-10 *(-571 C/A) genotype of CA and an *IL-4 *(-590 C/T) genotype of CT, there is important correlation with a *C3 *(1692 G/A) genotype of GG or AA (Figure 5b).

CysLTs, which are produced by inflammatory cells including eosinophils, are mediators of leukotrienes, and have been implicated in the pathogenesis of allergic diseases. Recently, it has been reported that CysLTs can act as autocrine or paracrine mediators to stimulate rapid, nonexocytotic release of IL-4 [19]. These findings are consistent with the present results, in which subjects with CT or TT genotype at *IL-4 *(-590 C/T), AG or GG genotype at *CysLT2 *(2534 A/G) and GG genotype at *IL-4Rα *(148 G/A) were estimated to be at high risk for pathogenesis of CAA (*P *= 0.00022). However, 2-SNP interaction between *CysLT2 *(2534 A/G) and *IL-4Rα *(148 G/A) (*P *= 0.00030) markedly affected the 3-SNP interaction.

In the present study, we examined correlation between CAA and 25 SNPs in 17 genes using an ANN model. We think that there are not a few main effects and interactions which can explain development of multifactorial disease like CAA, because it is thought that interactions of genetic risk factors might be different individually among CAA patients in spite of same disease. So it is very important to select multiple genetic factor models associated with multifactorial disease like CAA with high concentration ratio. We found that 10 of these SNPs are important factors in development of CAA. Important combinations among these 10 SNPs were also extracted. As described above, several of these combinations (listed in Table 6 etc.) have been found to be important factors in allergic disease, in previous biological and epidemiological studies. We also found several novel important combinations. The present data about important combinations suggests multiple patterns of CAA development. It should be noted that these findings were obtained automatically using an ANN model constructed without priori knowledge. Using an ANN model with 10 SNPs, we were able to discriminate between cases and controls with more than 70% accuracy. We concluded that the ANN is an effective tool for predicting development of CAA, using SNP data. However, further investigation of other genetic and environmental factors associated with CAA is needed. We previously constructed an advanced modeling method, the fuzzy neural network [20,21], which is an ANN model. When this model is applied to analysis, the susceptibility rules of interaction can be explicitly and linguistically described. Also, it can be used to describe susceptible interaction between genetic factors such as SNPs and environmental factors such as favorite foods and life style. Using the rules obtained with this model, we can plan protocols for preventive treatment of subjects with high-risk genetic profiles. Network analysis tools such as ANNs can be applied to analysis of multifactorial disease using SNP data such as selection of important SNPs or description of interactions between SNPs.

## Conclusions

Relationships between CAA and 25 SNPs in 17 candidate genes were analyzed using an ANN. In diagnostic prediction, ANN discriminated cases from controls more precisely than LR. From among the 25 original SNPs analyzed, we selected 10 SNPs that were closely associated with CAA. Calculating *P*-value using the χ^2 ^test, we found that 2-SNP and 3-SNP combinations of these 10 SNPs were associated with CAA. The ANN was able to represent associations between CAA and these 2-SNP or 3-SNP combinations using complicated nonlinear relations. Thus, the ANN can be used to characterize development of complex diseases caused by multiple factors.

## Methods

### Subjects and SNP data

SNP data were kindly provided by the ethics committees of Tohoku University and RIKEN. We analyzed the SNP data for 25 polymorphisms in the 17 genetic regions listed in Table 1. Each SNP was detected using the established method based on TaqMan PCR [22]. The study population comprised 172 subjects with childhood allergic asthma (CAA) who were under 17 years of age and 172 healthy subjects with no signs or symptoms of atopy-related diseases selected from general population, all of whom gave written informed consent for SNP analysis. The subjects were diagnosed by experienced doctors, as "positive" (with allergic asthmatic symptoms) or "negative" (without allergic asthmatic symptoms). In the present paper, the subjects with CAA are referred to as "cases" and the healthy subjects are referred to as "controls". Genotype patterns of the 25 SNPs were compared between cases and controls. None of the cases had genotype patterns coinciding with those of controls.

### Data preprocessing

To use SNP data as input data for the ANN, we converted the genotyping data into 2-numeral data. In ANN modeling, input and output variables are normalized into 0.1–0.9 [8]. In SNP data, there are 3 genotypes per locus. Therefore, we provided 2 inputs per SNP: (0.1, 0.1) for homozygote of the major allele, (0.1, 0.9) for heterozygote, and (0.9, 0.9) for homozygote of the minor allele. Since from the genetic point of view it may be difficult to estimate that heterozygote affects a disease by half the extent that homozygote affects it, the coding of (0.1), (0.5) and (0.9) was never used. The diagnosis data were also converted into numerical data, referred to hereafter as "teacher" values: 0.9 for "positive (case)", and 0.1 for "negative (control)".

For LR, we converted SNP data into numerical input data as follows: (0.1, 0.1) for homozygote of the major allele, (0.1, 0.9) for heterozygote, and (0.9, 0.9) for homozygote of the minor allele. Positive and negative diagnoses were also converted into numerical data: 0.9 and 0.1, respectively.

### ANN model and model construction

For SNP analysis, we used a three-layered ANN with input, hidden and output layers (Figure 1). For model construction, the performance index of the ANN was assessed using a method we previously proposed [7,8], with slight modifications. *N*_*error *_(number of missed points) and *Er *(sum of squared error) were defined and calculated for learning data and evaluation data as follows:


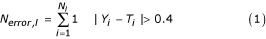



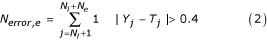


*N*_*error *_= *N*_*error*,*l *_+ *N*_*error*,*e *_    (3)


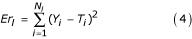



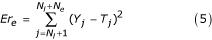



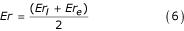


where *Y *and *T *represent the predicted value and the teacher value, respectively. *N*_*l *_and *N*_*e *_represent the number of subjects as learning and evaluation data, respectively. *N*_*error *_is the number of output data with an error of >0.4 between the predicted value and teacher value as shown above. *Er *is calculated with the square of error as shown above. For ANN learning, the connection weights were initially randomly set from 0 to 1, and were altered using the back propagation methods [23] with learning data so as to minimize the value of *Er*_*l*_. Learning rates of 0.1, 0.2, 0.3, 0.4 and 0.5 were examined. The maximum learning time was 2000 iterations. The best ANN model (selected for SNP analysis) was that in which *N*_*error *_reached minimum within the maximum learning time. When minimum *N*_*error *_was equal to that of other models within the maximum learning time, the model with minimum *Er *value was selected.

Prediction accuracy of the constructed model was defined as follows. Threshold was set at 0.5. If the teacher value was 0.9, and the predicted value was greater than 0.5, the prediction was true (true positive; TP); for predicted values lower than 0.5, the prediction was false (false negative; FN). If the teacher value was 0.1, and the predicted value was lower than 0.5, the prediction was true (true negative; TN); for predicted values greater than 0.5, the prediction was false (false positive; FP).

We calculated the prediction accuracy (*Ac*) as follows:


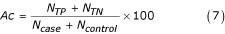


The sensitivity (*Se*) and specificity (*Sp*) of predicted values were defined as follows:


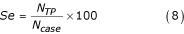



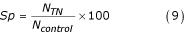


where *N*_*TP*_, *N*_*FN*_, *N*_*TN *_and *N*_*FP *_are the number of TN, FN, TN and FP subjects, respectively. *N*_*case *_and *N*_*control *_are the number of case and control subjects, respectively.

### Parameter Decreasing Method (PDM)

In order to extract SNPs closely associated with CAA, we selected the input variables by parameter decreasing method (PDM) after the ANN model with 25 SNPs was constructed. In PDM, 1 SNP was excluded from input variables in turn, and ANN models were constructed with the remaining 24 SNPs by performing the cross-validation described below. From among the 25 models thus constructed, the model with minimum *N*_*error *_averaged in the cross-validation step was selected. When minimum *N*_*error *_was equal to that of other models within the maximum learning time, the model with minimum *Er *value was selected as described above. The PDM step was repeated until 1 SNP remained as input variable. The PDM procedure was performed 5 times with unifying learning rates of 0.1 and learning time of 2000, and the rank of importance of selected SNPs was determined as described in Results section. We performed 5 PDM trials so that the effects of randomized initial connection weights might be minimized. In 5 PDM trials, data set for cross-validation mentioned below was reconstructed every time.

### Cross-validation

Cross-validation allows estimation of the prediction error of a model by leaving out a portion of the data as an evaluation data [24]. In the present study, to investigate the flexibility of the ANN, learning and evaluation were performed using the ANN and 5-fold cross-validation. With 5-fold cross-validation, the data set for the 172 cases and 172 controls was divided into 5 groups with randomizing and alternating the data. In each group, the number of cases was equal to that of controls. Four groups were assigned as learning data, and 1 group was assigned as evaluation data; this learning and evaluation process was repeated 5 times, so that each group was assessed once as evaluation data. Then, the prediction accuracy of evaluation data across all 5 trials was calculated and averaged for the overall prediction accuracy of the ANN model shown in Table 2. Sensitivity and specificity were also calculated.

### Logistic Regression (LR) Model

An LR model was constructed using SPSS 11.5J statistic software for Windows (SPSS Japan Inc., Tokyo), for comparison with the ANN model. All 25 SNPs were used as input variables of LR. For LR analysis, we used 50 main effects plus an intercept but not any interaction terms. As with the ANN model, the data set was divided into 5 groups and the cross-validation was performed. Prediction accuracy, sensitivity and specificity were calculated.

### Determination of differences in frequency of alleles and genotypes

We also examined association between CAA and combinations of SNPs by calculating *P*-value using a χ^2 ^test. The χ^2 ^test was used to evaluate the differences in frequencies of alleles or genotypes between cases and controls. The *P*-values shown in Table 1 were calculated using 172 cases and 172 controls. Degree of freedom (D.F.) (shown in Table 1) was 2 for 3 types of subjects; e.g., homozygote of the major allele, heterozygote, and homozygote of the minor allele. In the test with one SNP, when the expectancy for subjects homozygous for the minor allele (calculated from the frequency of the genotype) was less than 5 subjects for both case and control, we regarded the homozygote of the minor allele and the heterozygote as identical and defined degree of freedom as 1. In the tests with 2-SNP and 3-SNP combinations, we used the D.F. shown in Table 1 to find the change of differences in frequency under the same condition of SNP alone. If, in more than 5 subjects, all expectancies for subjects satisfied the test conditions, we calculated *P*-value with χ^2 ^test. In order to determine important combinations, we use two evaluation bases (*P*-value and effective combination value (ECV)) mentioned in Results section.

## Authors' contributions

YT carried out ANN modeling of SNP data including PDM and calculating *P*-value using a χ^2 ^test. ST and YH carried out the basic analysis using ANN and data preprocessing. YS and TS participated in providing of SNP data and the design of LR analysis. TK participated in the design of the study. HH conceived of the study, and participated in its design and coordination. All authors read and approved the final manuscript.

## Figures and Tables

**Table 1:** Polymorphisms used in the present study.

Gene	Polymorphism	D.F.^a^	*P*-Value^b^
*TGF-β*	-509 C/T	2	0.6299
*IL-10*	-571 C/A	2	0.1074
*IL-4*	-590 C/T	2	0.9085
*IL-4Rα*	148 G/A (Val50Ile)	2	0.0155
*TXA2R*	924 T/C (synonym)	1	0.5603
*ADRB2*	265 A/G (Arg16Gly)	2	0.0541
*STAT6*	2964 G/A	2	0.7881
*FcεRIB*	811 A/G (Glu237Gly)	1	0.5382
*ITK*	2860 G/A	2	0.9623
*CysLT2*	-580 T/C	2	0.7868
*CysLT2*	108 C/A	1	0.0036
*CysLT2*	2534 A/G	2	0.5664
*IL-12B*	1146 C/A	2	0.1154
*IKAP*	2446 A/C (Ile816Leu)	2	0.7988
*IKAP*	3214 T/A (Cys1072Ser)	2	0.2997
*IKAP*	3473 C/T (Pro1158Leu)	2	0.2779
*C*5	1155 A/G	2	0.1056
*C*5	4266 G/A	2	0.0581
*C*3	912 G/A	2	0.5020
*C*3	1692 G/A	2	0.6993
*C*3	4896 C/T	2	0.7205
*C5aR*	1289 C/A	2	0.1211
*C5aR*	1337 C/T	1	0.2398
*C3aR*	1526 G/A	1	0.7366
*IL-13*	329 G/A (Arg110Gln)	2	0.7924

^a^Degree of freedom (see text). ^b^Association between SNPs and CAA was evaluated as *P*-value, which was calculated with χ^2 ^test.

**Table 2:** Accuracy, sensitivity and specificity of ANN and LR.

a			
ANN	accuracy [%]	sensitivity [%]	specificity [%]

learning	98.8	99.1	98.4
evaluation	73.3	74.4	72.1


LR	accuracy [%]	sensitivity [%]	specificity [%]

learning	68.8	69.2	68.3
evaluation	48.3	45.3	51.2

b

ANN	accuracy [%]	sensitivity [%]	specificity [%]

learning	97.7	98.0	97.5
evaluation	74.4	77.9	70.9


LR	accuracy [%]	sensitivity [%]	specificity [%]

learning	59.4	57.8	60.9
evaluation	47.7	48.3	47.1

(a) with 25 SNPs.

(b) with 10 SNPs selected by PDM.

**Table 3:** Ranking of SNPs selected by PDM.

SNP	*P*-value^a^	n (/5)^b^	point (/50)^c^
*IL-4Rα *(148 G/A)	0.0155	5	47
*CysLT2 *(2534 A/G)	0.5664	5	38
*IL-10 *(-571 C/A)	0.1074	5	27
*C*3 (4896 C/T)	0.7205	4	27
*C*3 (1692 G/A)	0.6993	2	18
*IKAP *(2446 A/C)	0.7988	5	17
*IL-13 *(329 G/A)	0.7924	4	16
*C*3 (912 G/A)	0.5020	3	16
*STAT6 *(2964 G/A)	0.7881	2	14
*IL-4 *(-590 C/T)	0.9085	2	13
.........................................................................................................................................................................................................................................................			
*IKAP *(3473 C/T)	0.2779	2	12
*ADRB2 *(265 A/G)	0.0541	4	8
*IKAP *(3214 T/A)	0.2997	2	8
*C5aR *(1289 C/A)	0.1211	1	6
*C*5 (4266 G/A)	0.0581	1	5
*CysLT2 *(108 C/A)	0.0036	2	2
*C3aR *(1526 G/A)	0.7366	1	1

The 10 SNPs over the dotted line were used for the following experiments.

^a^*P*-value was calculated with χ^2^test.

^b^Number of SNPs selected within last 10 input variables during PDM procedure (5 trials performed).

^c^Point of SNPs selected within last 10 input variables during PDM procedure. The score of order ranged from 1 to 10 points, based on the significance order in 1 PDM procedure and totaled in 5 trials.

**Table 4:** Selection of effective combinations evaluated with two bases *P*-value and ECV among the 10 selected SNPs.

combination	2-SNP	3-SNP
*N*_*comb*_	90	360
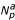	13	72
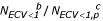	47/11	43/43
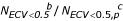	27/10	25/25
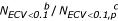	6/5	3/3

^a^The number of combination that satisfies the following conditions: *P*-value < 0.05.

^b^The number of combination that satisfies the conditions: ECV<1, 0.5 and 0.1, respectively.

^c^The number of combination that satisfies the conditions: both ECV<1, 0.5, 0.1 and *P*-value < 0.05, respectively.

**Table 5:** Number of effective combinations (*N*_*ef*_) and its concentration ratio.

2-SNP combination				
10 SNP^a^:15 SNP^b^	2 : 0	1 : 1	0 : 2	

*N*_*comb*_	90	300	210	
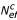	10	23	3	
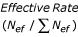	0.28	0.64	0.08	
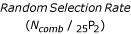	0.15	0.5	0.35	
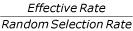	1.85	1.28	0.24	

3-SNP combination				

10 SNP^a ^: 15 SNP^b^	3 : 0	2 : 1	1 : 2	0 : 3

*N*_*comb*_	360	2025	3150	1365
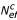	25	82	64	39
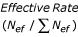	0.12	0.39	0.30	0.19
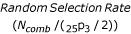	0.05	0.29	0.46	0.20
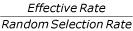	2.28	1.33	0.67	0.94

^a^Selected by PDM

^b^Not including 10 SNP selected by PDM

^c^The number of 2-SNP combination that satisfies the following conditions:*P*-value < 0.05 and ECV2<0.5.

The number of 3-SNP combination that satisfies the conditions: *P*-value < 0.05 and ECV3 < 0.5.

**Table 6:** Two-SNP interactions among the 10 selected SNPs (*P*-value < 0.05 and ECV2 < 0.5)

SNP 1	SNP 1 genotype	SNP 2	*P*^a^	*P*^b^	*P*^c^	*P*^b ^× *P*^c^	*P*^a^/(*P*^b ^× *P*^c^)
*C*3 (4896 C/T)	TT	*C*3 (1692 G/A)	0.01461	0.7205	0.6993	0.5038	0.0290
*CysLT2 *(2534 A/G)	AG+GG	*IL-4Rα *(148 G/A)	0.00030	0.5664	0.0155	0.0088	0.0344
*C*3 (1692 G/A)	AA	*C*3 (4896 C/T)	0.02858	0.6993	0.7205	0.5038	0.0567
*C*3 (912 G/A)	GG	*C*3 (4896 C/T)	0.02345	0.5020	0.7205	0.3617	0.0648
*C*3 (4896 C/T)	TT	*C*3 (912 G/A)	0.03073	0.7205	0.5020	0.3617	0.0850
*C*3 (1692 G/A)	GA	*CysLT2 *(2534 A/G)	0.04917	0.6993	0.5664	0.3961	0.1241
*STAT6 *(2964 G/A)	GG+GA	*IL-10 *(-571 C/A)	0.01752	0.7881	0.1074	0.0846	0.2070
*C*3 (4896 C/T)	TT	*IL-4Rα *(148 G/A)	0.00271	0.7205	0.0155	0.0112	0.2426
*C*3 (912 G/A)	GG	*IL-4Rα *(148 G/A)	0.00345	0.5020	0.0155	0.0078	0.4442
*IL-4 *(-590 C/T)	CT+TT	*IL-4Rα *(148 G/A)	0.00689	0.9085	0.0155	0.0141	0.4896

^a^*P*-value of combination of SNP 2 with genotype consisting of SNP 1. In this case, D.F. of SNP 2 is identical to that of Table 1.

^b^*P*-value of SNP 1 calculated alone.

^c^*P*-value of SNP 2 calculated alone.

^a^*P*^/(*b*^*P*^ × *c*^*P*^) represents effective combination value (ECV2).^
